# Supplementary material for: Transcriptome Analysis and GC-MS Profiling of Key Fatty Acid Biosynthesis Genes in Akebia trifoliata (Thunb.) Koidz Seeds
Source: Biology (Basel). 2022 Jun 3;11(6):855. doi: 10.3390/biology11060855 (PMC9220242; doi:10.3390/biology11060855)
Supplement: Supplementary file 1 [file biology-11-00855-s001.zip › Supplementary File(/Table S1.pdf]

Table S1.

Information of the FAD family in Arabidopsis thaliana (AtFADs) and Juglans regia L.(JrFADs).

Arabidopsis thaliana		Juglans regia L.	
Name	NCBI Reference	Name	NCBI Reference
AtADS1	NP_172098.1	JrADS3	XM_018974856.1
AtADS2	NP_001323798.1	JrALD	XM_019003847.1
AtADS3	NP_566529.1	JrDAL	XM_018979084.1
AtADS32	NP_188208.4	JrDES1-1	XM_018992767.1
AtADSL1	NP_172099.1	JrDES1-2	XM_018964647.1
AtADSL2	NP_172100.1	JrFAD2-1	XM_019004668.1
AtADSL3	NP_172102.1	JrFAD2-2	XM_019004667.1
AtADSL4	NP_172124.2	JrFAD2-3	XM_018993369.1
AtADSL5	NP_172125.1	JrFAD2-4	XM_018993367.1
AtDES1	NP_192402.1	JrFAD3-1	XM_018979912.1
AtSTAD7	NP_181899.1	JrFAD3-2	XM_018963672.1
AtFAD6	NP_001319529.1	JrFAD3-3	XM_018963671.1
AtFAD3	NP_180559.1	JrFAD3-4	XM_018963670.1
AtFAD4	NP_194433.1	JrFAD3-5	XM_018963669.1
AtFD4L1	NP_176410.1	JrFAD4	XM_018955731.1
AtFD4L2	NP_179874.1	JrFAD6	XM_018968019.1
AtFAD6C	NP_194824.1	JrFAD7	XM_018986723.1
AtFAD3C	NP_187727.1	JrFAD8-1	XM_018970364.1
AtFAD3D	NP_196177.1	JrFAD8-2	XM_018997622.1
AtSTAD1	NP_197128.1	JrSAD-1	XM_018983828.1
AtSTAD2	NP_186910.2	JrSAD-2	XM_018956614.1
AtSTAD3	NP_197127.1	JrSAD-3	XM_018956616.1
AtSTAD4	NP_186911.2	JrSAD-4	XM_018956617.1
AtSTAD5	NP_186912.1	JrSAD-5	XM_018962389.1
AtSTAD6	NP_175048.1	JrSAD6	XM_018993662.1
AtSLD1	NP_191717.1	JrSAD-6	XM_018963029.1
AtSLD2	NP_182144.1	JrSAD-7	XM_018962401.1
		JrSAD-8	XM_019001129.1
		JrSLD-1	XM_018989549.1
		JrSLD-2	XM_018980564.1
