# Supplementary material for: Transcriptome Analysis and GC-MS Profiling of Key Fatty Acid Biosynthesis Genes in Akebia trifoliata (Thunb.) Koidz Seeds
Source: Biology (Basel). 2022 Jun 3;11(6):855. doi: 10.3390/biology11060855 (PMC9220242; doi:10.3390/biology11060855)
Supplement: Supplementary file 1 [file biology-11-00855-s001.zip › Supplementary File(/Table S2.pdf]

Table S2

Sequencing data statistics

Samples	Clean reads	GC Content	%≥Q30	Mapped Reads
F1	23,256,659	44.72%	93.02%	43,146,935 (92.76%)
F2	20,685,251	44.78%	92.30%	38,142,251 (92.20%)
F3	22,572,069	44.46%	92.33%	40,685,068 (90.12%)
S1	24,386,978	44.77%	92.25%	44,801,673 (91.86%)
S2	24,701,298	44.60%	92.20%	45,102,572 (91.30%)
S3	26,568,263	44.60%	92.69%	49,047,004 (92.30%)
T1	22,435,026	44.45%	92.33%	40,573,326 (90.42%)
T2	28,941,826	44.62%	92.28%	52,466,624 (90.64%)
T3	24,861,869	44.68%	92.51%	45,097,534 (90.70%)
U1	20,954,369	44.59%	91.91%	37,878,449 (90.38%)
U2	26,632,118	44.56%	94.83%	48,787,640 (91.60%)
U3	21,747,198	44.63%	93.95%	39,688,044 (91.25%)
I1	21,867,427	44.18%	94.04%	40,179,045 (91.87%)
I2	25,370,936	44.00%	93.14%	46,274,457 (91.20%)
I3	27,432,544	44.19%	93.56%	50,484,174 (92.02%)
