# Supplementary figures and images for: Transcriptome Analysis and GC-MS Profiling of Key Fatty Acid Biosynthesis Genes in Akebia trifoliata (Thunb.) Koidz Seeds
Source: Biology (Basel). 2022 Jun 3;11(6):855. doi: 10.3390/biology11060855 (PMC9220242; doi:10.3390/biology11060855)

A

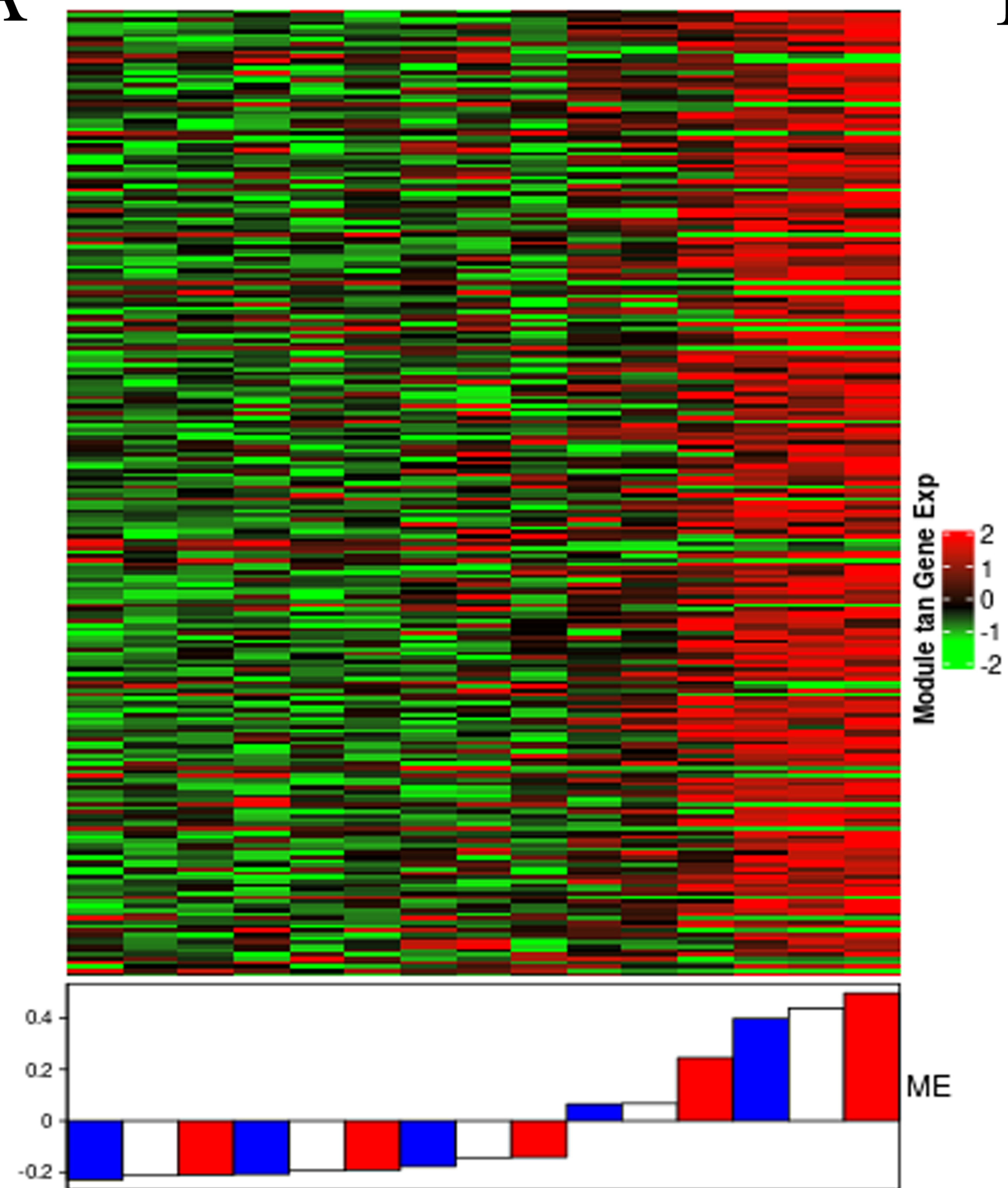

B

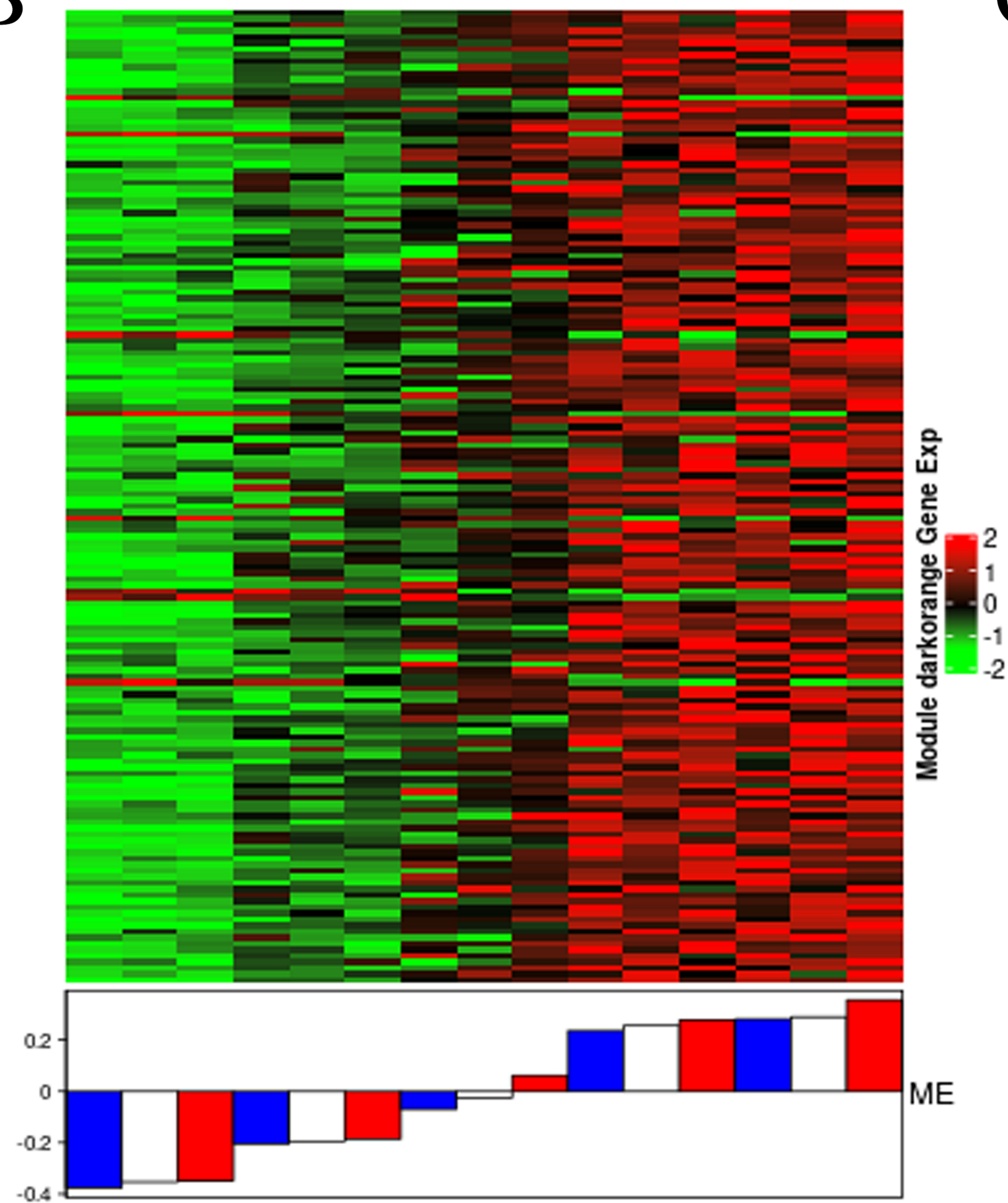

C

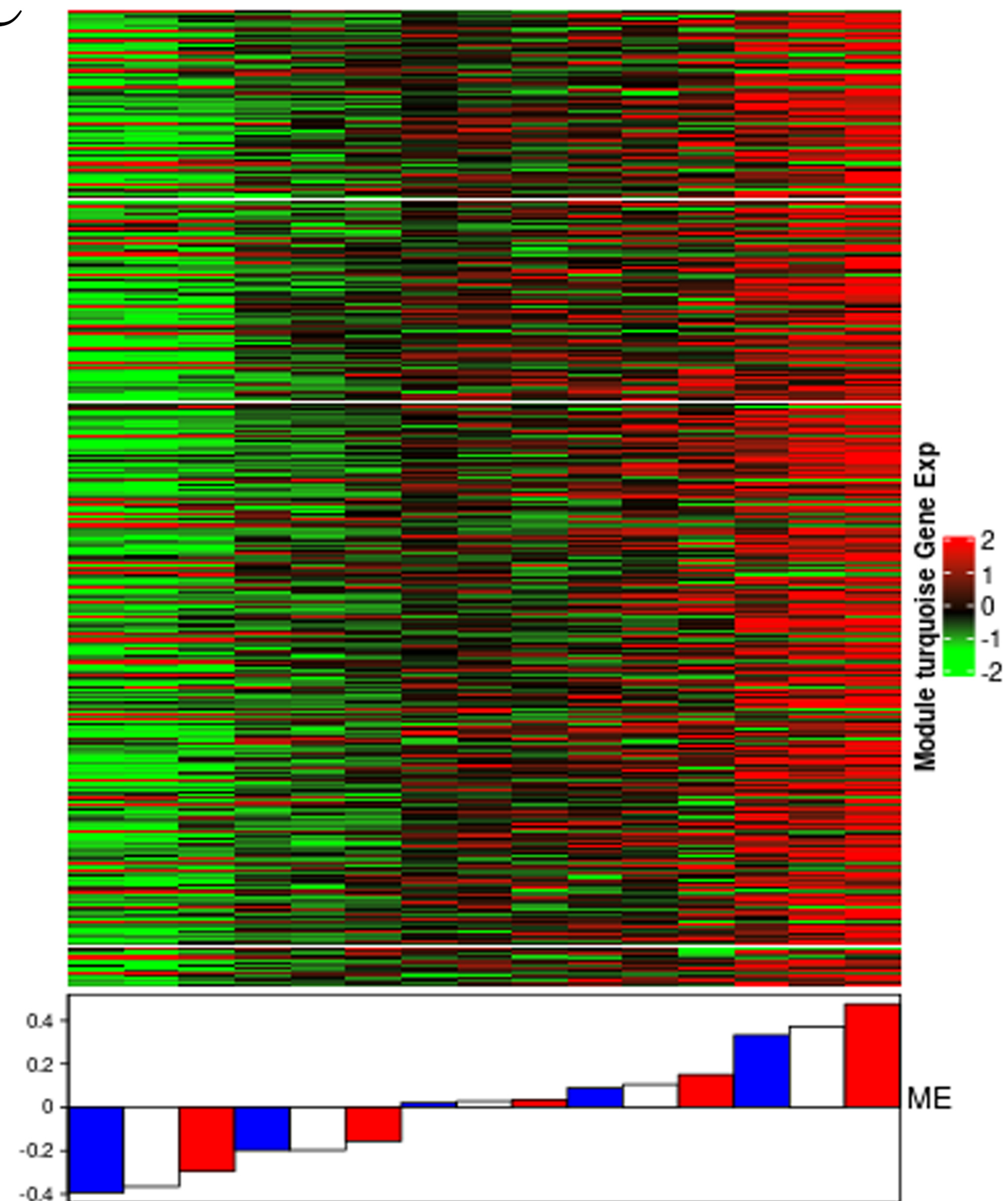

Supplement: Supplementary file 1 [file biology-11-00855-s001.zip › Supplementary File(/Figure S1.pdf]
