# Supplementary material for: Transcriptome Analysis and GC-MS Profiling of Key Fatty Acid Biosynthesis Genes in Akebia trifoliata (Thunb.) Koidz Seeds
Source: Biology (Basel). 2022 Jun 3;11(6):855. doi: 10.3390/biology11060855 (PMC9220242; doi:10.3390/biology11060855)
Supplement: Supplementary file 1 [file Figure S2.pdf › Supplementary File(/Figure S2.pdf]

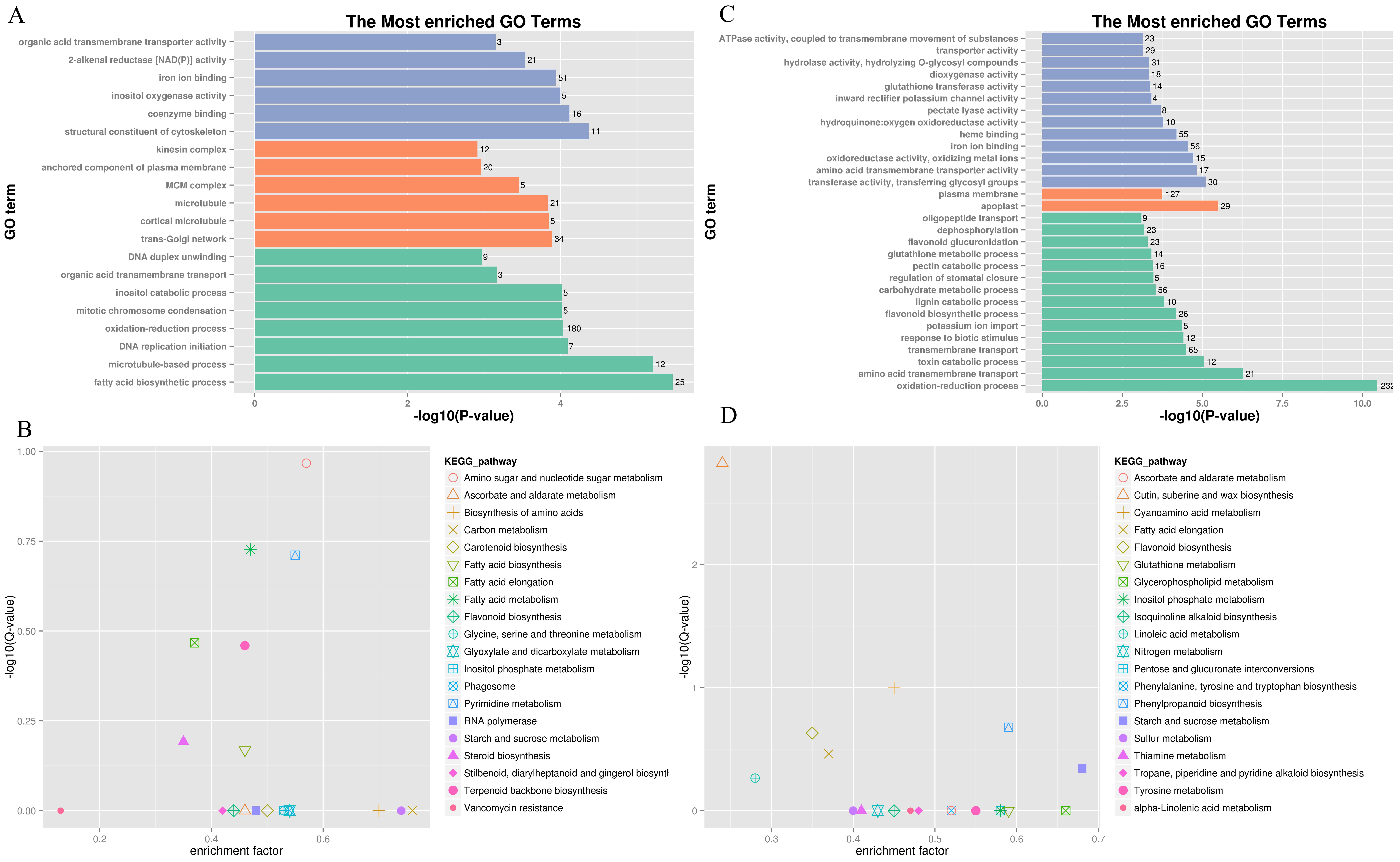

A
The Most enriched GO Terms
GO term
organic acid transmembrane transporter activity 3
2-alkenal reductase [NAD(P)] activity 21
iron ion binding 51
inositol oxygenase activity 5
coenzyme binding 16
structural constituent of cytoskeleton 11
kinesin complex 12
anchored component of plasma membrane 20
MCM complex 5
microtubule 21
cortical microtubule 5
trans-Golgi network 34
DNA duplex unwinding 9
organic acid transmembrane transport 3
inositol catabolic process 5
mitotic chromosome condensation 5
oxidation-reduction process 180
DNA replication initiation 7
microtubule-based process 12
fatty acid biosynthetic process 25
0
2
4
-log10(P-value)
C
The Most enriched GO Terms
GO term
ATPase activity, coupled to transmembrane movement of substances 23
transporter activity 29
hydrolase activity, hydrolyzing O-glycosyl compounds 31
dioxygenase activity 18
glutathione transferase activity 14
inward rectifier potassium channel activity 4
pectate lyase activity 8
hydroquinone:oxygen oxidoreductase activity 10
heme binding 55
iron ion binding 56
oxidoreductase activity, oxidizing metal ions 15
amino acid transmembrane transporter activity 17
transferase activity, transferring glycosyl groups 30
plasma membrane 127
apoplast 29
oligopeptide transport 9
dephosphorylation 23
flavonoid glucuronidation 23
glutathione metabolic process 14
pectin catabolic process 16
regulation of stomatal closure 5
carbohydrate metabolic process 56
lignin catabolic process 10
flavonoid biosynthetic process 26
potassium ion import 5
response to biotic stimulus 12
transmembrane transport 65
toxin catabolic process 12
amino acid transmembrane transport 21
oxidation-reduction process 232
0.0
2.5
5.0
7.5
10.0
-log10(P-value)
B
-log10(Q-value)
1.00
0.75
0.50
0.25
0.00
0.2
0.4
0.6
enrichment factor
KEGG_pathway
Amino sugar and nucleotide sugar metabolism
Ascorbate and aldarate metabolism
Biosynthesis of amino acids
Carbon metabolism
Carotenoid biosynthesis
Fatty acid biosynthesis
Fatty acid elongation
Fatty acid metabolism
Flavonoid biosynthesis
Glycine, serine and threonine metabolism
Glyoxylate and dicarboxylate metabolism
Inositol phosphate metabolism
Phagosome
Pyrimidine metabolism
RNA polymerase
Starch and sucrose metabolism
Steroid biosynthesis
Stilbenoid, diarylheptanoid and gingerol biosynt
Terpenoid backbone biosynthesis
Vancomycin resistance
D
-log10(Q-value)
2
1
0
0.3
0.4
0.5
0.6
0.7
enrichment factor
KEGG_pathway
Ascorbate and aldarate metabolism
Cutin, suberine and wax biosynthesis
Cyanoamino acid metabolism
Fatty acid elongation
Flavonoid biosynthesis
Glutathione metabolism
Glycerophospholipid metabolism
Inositol phosphate metabolism
Isoquinoline alkaloid biosynthesis
Linoleic acid metabolism
Nitrogen metabolism
Pentose and glucuronate interconversions
Phenylalanine, tyrosine and tryptophan biosynthesis
Phenylpropanoid biosynthesis
Starch and sucrose metabolism
Sulfur metabolism
Thiamine metabolism
Tropane, piperidine and pyridine alkaloid biosynthesis
Tyrosine metabolism
alpha-Linolenic acid metabolism
